# Living an ordinary life – yet not: the everyday life of children and adolescents living with a parent with deafblindness

**DOI:** 10.1080/17482631.2022.2064049

**Published:** 2022-04-18

**Authors:** Karina Huus, Ann-Sofie Sundqvist, Agneta Anderzén-Carlsson, Moa Wahlqvist, Maria Björk

**Affiliations:** a Department of Nursing Science, Jönköping University, Jönköping, Sweden; b Faculty of Medicine and Health, University Health Care Research Center, Örebro University, Örebro, Sweden; c Audiological Research Center, Faculty of Medicine and Health, Örebro University, Örebro, Sweden; d The Swedish National Resource Center for Deafblindness, Lund, Sweden

**Keywords:** Child as a relative, deafblindness, everyday life, parent, participation

## Abstract

**Introduction:**

The family life of people living with one family member with deafblindness has been sparsely described.

**Purpose:**

The aim of the study was to explore how children experience their everyday family life when having a parent with deafblindness.

**Methods:**

An explorative study in which data have been collected by qualitative interviews of children. Qualitative content analysis has been used for analysing the data.

**Results:**

Overall theme; Living an ordinary life—yet not, is based on four categories with subcategories. *A family like any other* describes: Having the same family life as their friends, Acting like other children and It is what it is. *Different everyday life* describes: Acknowledging differences, Adjusting to the parent’s needs and Financial strain. *Being there for the parent* describes: Helping the parent and Protecting the parent from harm. *Being emotionally affected* describes: Feelings of frustration, Feelings of compassion and Need for support.

**Conclusion:**

Children as relatives of parents with deafblindness have been given a voice. The children live an ordinary life, but at the same time a different ordinary life. Professionals need to take the child and their needs into account when support is given.

## Introduction

This study aims to explore how children experience their everyday family life when having a parent with deafblindness. Deafblindness is a highly diverse low-incidence disability which is estimated to affect 0.2–2% of the world population (WFDB, 2018). In the Nordic definition, deafblindness is defined as a distinct disability which consists of a combined hearing and vision impairment of such a degree that the impaired senses fail to compensate for each other (Nordic Welfare Centre, 2016). Deafblindness has many causes, and the degree of hearing and vision impairment is very varied, meaning that the individual´s experiences of the consequences of deafblindness can be vast (Larsen & Damen, 2014; Moller, 2003). In Sweden, where this study was conducted the estimated number of people living with deafblindness under the age of 65 is 2000 (Nkcdb, 2021).

Previous research has found that people with deafblindness encounter difficulties with communication, participating in activities, mobility and being independent (Fletcher & Guthrie, 2013; LeJeune, 2010). Restrictions in health-related quality of life and well-being have also been described (Wahlqvist et al., 2013, 2016) as well as fear of what the future will bring (Fletcher & Guthrie, 2013; LeJeune, 2010).

The family life of people with deafblindness has been sparsely described from the perspective of the individual with deafblindness, reporting on needs of support both in emotional and practical aspects that are met or unmet by family members (Ellis & Hodges, 2013; LeJeune, 2010; Miner, 1995, 1997). The partner perspective of someone with deafblindness, mainly described for an ageing population, has highlighted challenges in communication that affect well-being (Lehane, Dammeyer, Elsass et al., 2017; Lehane, Dammeyer, Hovaldt et al., 2017; Lehane et al., 2018).

Two recently published articles have described the health-related quality of life, family climate and sense of coherence in families where a parent has deafblindness, as well as the partner’s (other parent) perspective on parenting with a parent with deafblindness (Björk et al., 2022; Wahlqvist et al., 2020). Wahlqvist et al. (2020) quantitatively described how the health-related quality of life was affected for all family members, with the highest risk for a poor outcome for the parent with deafblindness. Closeness was described by the family members as one of the core emotional aspects of family life. Qualitative interviews were conducted with partners of parents with deafblindness, who said that the consequences of deafblindness ruled the family, and the partner’s needs were overlooked (Björk et al., 2022).

There is previous literature in the area of the parent having a disability. Earlier studies have predominately focused on negative aspects of being a child of a parent with a disability (Jacob et al., 2019; Kirshbaum & Olkin, 2002). This discourse is changing and research addressing assets and positive outcomes indicates a more complex situation (Krauss & Olkin, 2020). However, problems that have been identified are that these children and adolescents can experience that their everyday life with their friends in school and relationships within the family can be negatively impacted (Pakenham & Cox, 2012; Pedersen & Revenson, 2005; Rolland, 1999; Sieh et al., 2010). They also need to support their parents in doing household tasks (Damen et al., 2022; Thomas et al., 2003) and they experience life changes including negative personal and relational impacts (Faugli et al., 2020). However, Aldridge and Becker (1999) emphasized that children, regardless of whether they grow up with a parent who has a disability or not, will care for and about others as a part of development from child to adult. This means helping with household chores, taking care of siblings and more. Nevertheless, they argue problems might occur if the child is expected to take on responsibilities to such an extent that his or her own development is at risk.

Eden et al. (2017) addressed being a child of parents with sensory impairment (vision or hearing respectively). The results showed that the emotional competence as well as empathy was significantly higher in children and adolescents who had a parent with either a visual or hearing impairment than in those who had a parent without a disability. Duvdevany et al. (2007) compared adolescents of parents with visual impairments with parents without a vision impairment. The result highlighted similarities between the two groups regarding free time, anxiety and feeling towards the parent. Differences were found in terms of a richer social life and better quality relationships with friends, where the children with a parent with a visual impairment had better outcomes.

Research on children and adolescents with deaf parents has shown that these children have a shared language (sign language) and culture with the parent within the Deaf community as well as in the hearing community (Fox, 2018). To be deaf is not restricted to a medical condition based on audiological findings; it is rather defined as belonging to a cultural and linguistic minority in the hearing society often referred to as “Deaf” with a capital “D” (Fox, 2018; Singleton & Tittle, 2000). Children and adolescents with deaf parents are sometimes abbreviated as Coda/Kodas and often through their double cultural identity mediate between the Deaf and hearing society by being interpreters and spokespersons for the family in interactions with the surrounding society (Singleton & Tittle, 2000). In a study by Knight (2018) the children (i.e., Kodas) expressed feelings of belonging, responsibility and appreciation for their deaf parents. They also felt otherness in relation to school or in public, stating that they were sometimes viewed as “awkward” if their identity as a Koda was known.

It has been shown that children are affected when a parent has a disability, however this has yet not been studied for children who have a parent with deafblindness. The purpose of the present study was to explore how children and adolescents experience their everyday family life when having a parent with deafblindness.

## Material and methods

### Design

This explorative descriptive study reports the analysis of data collected by qualitative interviews of children and adolescents of parents with deafblindness. This study is a part of a larger research project focusing on exploring health and family climate in families where one parent has deafblindness (Björk et al., 2022; Wahlqvist et al., 2020).

### Participants

A convenience sampling technique was used. Included in the study were 18 children from 12 families: 14 girls and four boys. Their ages ranged between 5 and 19 years (mean 11 yrs.). One of the included children had a hearing loss. As their first language, seven of the children reported spoken language, six visual sign language and five reported mixed languages (both spoken and sign language). They used different everyday languages; the majority used spoken language or mixed language, depending on with whom they talked. All participants in this study are hereafter referred to as children, regardless of their age.

### Procedure

The board members of the family section of the non-governmental organization The Association of the Swedish Deafblind (FSDB) shared information about the entire project both verbally and on Facebook. Information was also offered at gatherings by the FSDB, where the oral information was interpreted into sign language by professional interpreters. Parents of eligible children shared this information with the children. After being given time to ask questions and reflect on their participation, the older children consented and the parents signed informed consent forms for the younger children. In addition, the children themselves provided a written assent.

### Data collection

The children were individually interviewed by researchers experienced in interviewing children (KH, AAC & MB). As all children spoke Swedish, the interviews were conducted using spoken language and employing a semi-structured interview guide. The children decided the time and place for the interview. The interview began with the open question “Please, tell me what it is like to have a parent with deafblindness!” Further questions revealed how the children experienced how the parent’s deafblindness impacted family life. The interviewers aimed at covering all the areas in the interview guide in all interviews. The areas were: daily life, including situation at home, school and leisure activities, impact of the deafblindness on the family situation, the strengths of the parent with deafblindness, family roles and need for support for the child and/or family. In addition, the children were asked to speak about two situations where the deafblindness had a positive and negative impact, respectively. Using remarks such as “Please, tell me more!” “How do you mean?” were used to help the child to express themselves more fully. The interviews were adapted to the age and development of the child. If the child for example, did not understand a question, it was rephrased with words that was easier for the child to understand. If needed the child could take a break during the interview, which however was not necessary at any occasion. The interview lasted 10–48 minutes (mean 24 min). All interviews were audio-recorded and transcribed verbatim. In one interview an interpreter was present and, in another interview, both an interpreter and a parent were present.

### Analysis

Data was analysed with conventional qualitative content analysis as described by Hsieh and Shannon (2005). The researchers read through the transcripts several times to get a sense of the whole. The second author (ASS) developed an initial coding framework by reading the transcripts word by word, and highlighting exact words that appeared to capture key thoughts. The codes were sorted into subcategories based on their similarities, building meaningful clusters (see, Table 1 for examples of the analysis process).

**Table 1.** Examples of exact words, codes, subcategories and categories

Exact words	Code	Subcategory	Category
Both my mum and my father are there for me [regardless of having deafblindness or not]	Parental support	Having the same family life as their friends	A family like any other
Being in the skate park, hanging out with friends, or playing games	Leisure time activities	Acting like other children	
I have never cared about what other people think	Don’t care	It is what it is	
If we are going to take a spontaneous trip then we have to call a cab or check the bus timetable	Different family	Acknowledging differences	Different everyday life
Don’t walk at a fast pace since my parent won’t be able to keep up	Adapt the pace	Adjusting to the parent’s needs	
We don’t have everything like everyone else	Don’t have everything	Financial strain	
I need to be my parent’s eyes and ears and explain things to my parent	Connector to the surrounding world	Helping the parent	Being there for the parent
Looking out for my parent so they doesn’t hurt themselves	Keeping an eye on	Protecting the parent from harm	
When I say they [the parent] cannot hear, they [other people] start speaking English or with a loud voice close to my parents face as if my parent does not understand anything	Other people’s ignorance	Feelings of frustration	Being emotionally affected
You should never think that deafblind people have it so easy. They have a really hard time	Having a hard time	Feelings of compassion	
Support from my sister, she has “experienced” the same things as me	Support from siblings	Need for support	

Then categories were developed by gathering subcategories together. To assure quality, AAC independently derived codes and subcategories from a few randomly chosen transcripts. Differences and congruencies were discussed between these two authors. The final analysis was discussed within the whole research group until consensus regarding the subcategories, categories and the overarching theme was reached.

### Ethical considerations

The Regional Research Ethics Board Uppsala, Sweden gave formal approval of the study (2016/124). The study was conducted in accordance with the ethical principles of respect for autonomy, non-maleficence, beneficence, and justice as stated in The Declaration of Helsinki. The ethical principles were considered throughout the study. All participants received written and oral information about the study and were given opportunity to ask questions. All participants were informed about confidentiality and the right to withdraw at any time without explanation. After been given time for consideration, parents and children older than 15 years gave their written informed consent and children under the age of 15 provided their written or verbal assent. People with deafblindness constitute a small and rare group in society. By adding the requirement of having a family and children, the possible number of participants in Sweden becomes even smaller. Therefore, details about the children’s and families’ characteristics are sparse to protect their privacy.

It is important to give children a voice and include them in research (Nilsson et al., 2015). In order for the child to feel safe, they could choose if they wanted to have a parent present during the interview, which one child chose. Before the interview the researchers tried to spend some time with the child in order to build a rapport (Irwin & Johnson, 2005).

## Results

**Figure 1.**
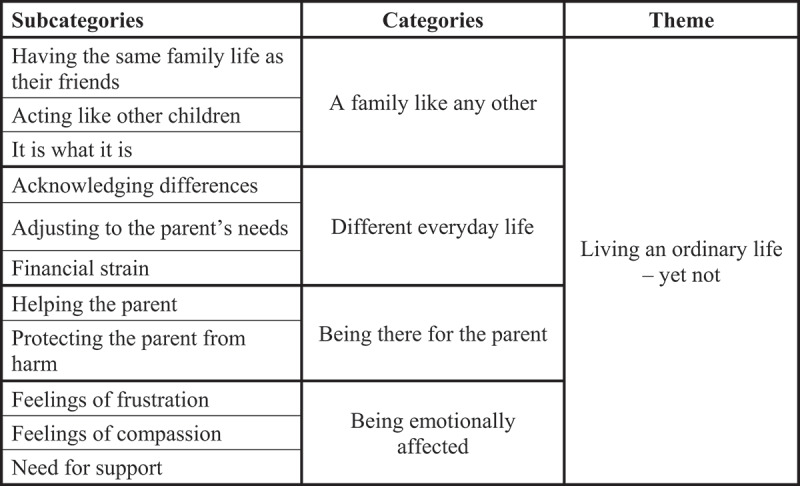
Overview of the subcategories, categories and theme.

During the analysis 11 subcategories, four categories and one unifying theme were identified. The theme, *Living an ordinary life—yet not*, was interpreted as the core of how children experienced their everyday life living with a parent with deafblindness, covering the content of the subcategories and categories. The first part of the theme, i.e., *Living an ordinary life*, relates to the children’s descriptions of being part of a family like any other whereas the second part,—*yet not*, describes their experiences of a somewhat different everyday life. An overview of the theme, categories and subcategories is shown in Figure 1.

## A family like any other

Living with a parent with deafblindness was described by all children as having a family like any other. This category is characterized by *Having the same family life as their friends, Acting like other children* and *It is what it is*.

### Having the same family life as their friends

All children living with a parent with deafblindness said they had the same family life as their friends. They said that there was nothing especially good or bad about having so. Instead, there were both positive and negative things connected to this and the deafblindness did not affect the child’s family life. One child stated:

*There is nothing we can’t do because my parent has deafblindness. My family does the same things as my friends' [families of their friends]*. (Interview no 17)

Having the same family life as their friends included for example, going grocery shopping with the parent or that the parent went shopping by themselves. The children said that they watched television or played games with their parents in their spare time. Other things interpreted as showing they had the same family life as their friends varied between the children, but could include that the family went on vacations, doing the same things as their friends, having pets, that both of their parents attended meetings at school, and that their parent was independent and managed to run their everyday life by themselves. In most families, the entire family helped out with tidying up, and some of the children mentioned doing the dishes/laundry or cooking. The meals prepared by the parents were described as equally good, regardless of whether the parent had deafblindness or not.

### Acting like other children

The children described that they did the same activities as other children such as playing with their friends, riding a bike, listening to music, watching movies, playing the piano or being in their room reading a book and one of them described it like *“they [my friends] do the same things as I do”* (Interview no 6). Some of the children said that they, just like their friends, did not practice any sports, or that they did not participate in any leisure time activities. The children said that they did not talk so much with their parents regarding their personal problems; instead, they talked with their friends, they spent time with their friends at home or at their friends’ homes. Furthermore, they wanted to be treated like anyone else, that is in the same way like any one of their friends.

### It is what it is

The parent’s deafblindness was a natural part of the children’s daily life and the parent’s disability was described as an integral part of their lives. The deafblindness was not reflected upon as something peculiar by the children and it was described as *“nothing special*” (Interview no 5), *“it’s nothing”* (Interview no 14), *“I’m used to it”* (Interview no 12), *“nothing weird or different”* (Interview no 7), and *“it is what it is”* (Interview no 14). The children stated that their friends knew, or at least that they thought that their friends knew, that their parent had deafblindness. Since having a parent with deafblindness was a natural part of the children’s lives this was something they did not always tell their friends since they did not think they would have to. This was instead something their friends eventually found out by themselves.

## Different everyday life

Although the children stated that they lived a life like anyone else, they also said that they were experiencing a different everyday life. This included *Acknowledging differences*, that they were *Adjusting to the parent’s needs*, and that the parent’s deafblindness led to a *Financial strain*.

### Acknowledging differences

Almost every child stated that their everyday life in some aspects differed from those of their friends. The differences connected to having a parent with deafblindness were described in both positive and negative wordings. The positive differences included that they were allowed to skip the queue when going to amusement parks, could take a taxi for a small amount of money or go to special camps for families with deafblindness and meet with friends living under similar conditions. Some of the children said that they liked having a parent that was considered different and that they themselves liked being special/different. On the negative side was the fact that the parent did not have a driving licence, resulting in that it took a lot of planning if the family wanted to go somewhere.

Strolling down the town with the parent could also be a negative experience, since other people might look at them, pointing and commenting. Some of the parents had a guide dog and this was experienced as both positive and negative by the children. Positive since the dog had relieved the burden for others in the family a little by making the parent with deafblindness more independent. One child (Interview no 11) said that *“it [the dog] is my parent’s eyes”*, and that *“it [the dog] is helping my parent”*. The negative emotion connected to the guide dog was that the dog drew attention to them, which was also reported if the parent used a cane while walking.

Some of the children stated that they had to take on an adult role in order to manage their everyday life due to the parents’ deafblindness, as described by one child below

*I had to take on so much responsibility at home, so I was already like an adult at home with my parent all the time I was there. I was the one who always cleaned the house or I mean the apartment; it was me who cooked the food and did the laundry and the dishes and everything. It made me have an adult role when I was with other kids and they didn’t understand me. They didn’t understand me, because I knew they liked to run around and play, but I never did that when I was a kid. Well of course I did, but I didn’t understand it and it was very frustrating and confusing and stuff like that*. (Interview no 8)

A few of the children talked about school and doing homework. They said that they had difficulties managing school since their parent could not give them the same help with their homework as their friends got from their parents.

### Adjusting to the parent’s needs

Some of the children said that they needed to adjust themselves to their parent’s needs, as stated by one child *“I have always adapted myself to my parents’ needs”* (Interview no 7). Adjusting to the parent’s needs included the need for adapting the communication method. The children described that they adapted the communication method in accordance with the parent’s needs, and they said that they sometimes communicated by spoken language and sometimes by sign language. One child described using spoken language when communicating with a sibling, but both used sign language when communicating with their parents.

*we [my sister and I] use sign language when sitting down at the dinner table with our parents, but we use spoken language if we sit there by ourselves*. (Interview no 15)

Some of the children stated that when the parent was attending meetings, the theatre, or graduation at school a professional interpreter was always present.

The children also described that they had to put things back in their predetermined places so that the parent would be able to find them without seeing, creating a sense of security for the parent. One child had to move to another city because the parent wanted to live close to his/her own mother when the family situation changed.

### Financial strain

Having a parent with deafblindness might lead to financial strain for the children. One child said that one of the parents had to work a lot of overtime to survive financially since the other parent was unemployed due to deafblindness, and one child said that there were a *“lot of expenses involved when being deafblind”* (Interview no 14). Another child said that the parents had taken a music instrument for repair and that they could not afford to pay for it when it was mended, which made the child sad, as s/he liked to play the instrument.

## Being there for the parent

Being the child in a family where at least one of the parents had deafblindness meant being there for the parent by *Helping the parent* and *Protecting the parent from harm*.

### Helping the parent

Helping the parent included many things and was something many of the children said that they wanted to do. It could involve things like helping the parent with shopping for groceries, finding things at home, being the parent’s eyes by guiding the parent, interpreting or informing other people that *“my parent has deafblindness so that is why s/he did not see you and therefore bumped into you”* (Interview no 12). Helping the parent also included being the parent’s eyes when shopping for clothes, or acting as a counsellor, listening to and supporting the parent.

*Often my parent [with deafblindness] feels bad, and usually in our family no one wants to talk to my parent then because s/he just talks negatively and it is a bit difficult. So, when my parent feels bad then s/he comes to me and talks a little. And I act as my parent’s ‘counsellor.’* (Interview no 1)

### Protecting the parent from harm

Some of the children described a willingness and preparedness to protect their parent from harm. This was done by keeping the home tidy and packing away their toys if they had been playing, as stated by one of the children *“I make sure that there are no toys in the way since this might cause my parent to trip”* (Interview no 16). But it also involved the children putting themselves in front of the parent to prevent them from tripping when out for a walk, or informing the parent that something was blocking the way so that the parent could go around it.

Protecting the parent from harm also involved making sure that the parent was using their white cane when out walking, or assisting in putting on a [reflective] vest when going skiing for example.

## Being emotionally affected

*Feelings of frustration, Feelings of compassion* and *Need for support* were the subcategories interpreted as describing how the children were emotionally affected by living with a parent who had deafblindness.

### Feelings of frustration

Since deafblindness affects both the person’s sight and hearing the children said that there could be misunderstandings about different situations, which in turn led to frustration. Such a situation was described by one child when doing homework. As the parent could not clearly see what was happening, they sometimes accused the child of playing with his/her tablet instead of doing the homework. This accusation made the child frustrated since s/he actually was doing the homework. Other situations when feelings of frustration occurred were when the children had to repeat themselves several times since their parent did not hear what was being said, or having difficulties being patient since everything took much more time when having a parent with deafblindness, for example, shopping at the mall or when taking a family trip.

Most of the frustration was, however created in relation to other people who did not understand what deafblindness implies. It could be the cashier at the grocery shop who did not know how to approach the parent, or when other people reacted with fear of the parent, possibly due to their own uncertainty of deafblindness. There was a thin line between frustration created by the ignorance regarding deafblindness from other people, and the frustration created when other people asked too many questions when trying to understand how the parent was affected by the deafblindness. The insensitivity from others also caused frustration.

*Perhaps it is not appropriate to tell me that you would commit suicide if you were deafblind. Others have actually told me that many times: ‘but Gosh what a pity that your parent is deafblind. If I were deafblind I would have committed suicide a long time ago’. This makes me feel very uncomfortable and really sad and I think: ‘Does my parent want to commit suicide or is s/he doing well?’* (Interview no 11)

### Feelings of compassion

Having a parent with deafblindness created feelings of compassion among the children. The children said that they felt sorry about their parent’s inability to see and hear, and that they described what they saw or heard for the parent in order to facilitate for a shared picture of the reality. It could for example, be a description of the colour of the sky, the patterns on the wings of a butterfly, or the sound of birds singing in the trees. Some of the children said that they perceived that their parent was excluded due to the deafblindness, leaving them lonely and almost without friends.

The children experienced that the deafblindness helped them become close to their parent and family, and they enjoyed spending time with each other. One child stated that

*I often go to town with my parent and this is not usual among other teenagers. They do not want to go with their parent, but I think it is nice. Just because s/he may not be able to go to a clothing store and find things s/he needs, so then we can go shopping together*. (Interview no 14)

### Need for support

Some of the children stated that they had not been offered any special support in school or from a counsellor due to their parent having deafblindness. Since some of the children stated that they believed that they had difficulties managing school due to the lack of help from their parent with their homework they believed that they would have benefitted from some special help in school. Despite this, some stated that they did not need any special support and some children expressed a need for emotional support; not only for themselves but also for other children experiencing the same situation as themselves. The present emotional support was mostly given by family and friends, *“I get the support I need from my friends”* (Interview no 5), and in some cases from the school, *“I´ve talked a little with the school nurse regarding how it is to have a parent with deafblindness.”* (Interview no 9).

## Discussion

This is, to the best of our knowledge, the first study with the aim of describing how children and adolescents experience their everyday family life when having a parent with deafblindness. The main theme, *Living an ordinary life- yet not*, shows that the children experience that their family is like any other family, although having a somewhat different everyday life, which at times affects the children both positively and negatively. This is in line with previous studies describing experiences of children of parents with various disabilities (Jacob et al., 2019; Kirshbaum & Olkin, 2002; Thomas et al., 2003). The fact that a parent’s disability affects the entire family in different ways (Kirshbaum & Olkin, 2002; Olkin et al., 2006; Wahlqvist et al., 2020), was confirmed in the present study.

The overall message of the narratives was that the children viewed themselves as living an ordinary life, like their friends, although they could identify some differences. This view could be supported by a previous study, where these children rated their health-related quality of life as similar to a reference population (Wahlqvist et al., 2020). One positive reflection on being a child of a parent having deafblindness was that the children felt that they had something special within their family, that they were unique. This feature has previously been described by Knight (2018), regarding children of deaf parents. In the present study, the children mentioned the benefit of being bilingual, and they were proud of being able to communicate with people with deafblindness, but this was not as pronounced as in the study by Knight (2018) where hearing children of deaf parents experienced that they were part of two cultures.

The children mentioned that they were emotionally affected by having a parent with deafblindness, which included both positive aspects, as well as negative. The parent’s disability created a closeness between the child and the parent. This was also identified in the study by Wahlqvist et al. (2020), where family members individually rated closeness as a significant aspect of family climate. In the study by Knight (2018) the children whose parents were deaf, expressed feelings of belonging, responsibility and appreciation for their deaf parents, which is somewhat like the positive aspects of being emotionally affected identified in the present study. This is in line with Eden (2017) who identified developed emotional competence and empathy in children of parents with sensory impairment. Nevertheless, young carers (9–24 yrs) in Australia, who had a caring responsibility for a relative with an illness or disability reported a need for some respite from responsibilities, being able to join social activities (Moore & McArthur, 2007). However, in the present study children said that they spent time with their friends, both at home and at their friends’ homes, although there were also some exceptions to this picture.

In the present study some children said that they needed to be more involved and help with household tasks, for example, to clean, do the dishes and laundry which sometimes could be experienced as a hindrance to playing with other children, and they said they sometimes felt like an adult within their family. Thomas et al. (2003) found that children of parents with illness or disabilities have to take a somewhat greater responsibility in the household. The child’s quality of life can be affected by the amount of responsibility they have to take at home and on how much support the child receives from other adults (Aldridge & Becker, 1999; Kallander et al., 2020). Therefore, it is important to support the entire family, both the parent with disability and children, in order to be able to live a healthy life and develop as a family (Aldridge & Becker, 1999).

It is necessary to provide support to children as next of kin with regard to their situation or preferences (Steffenak et al., 2021). For this specific group of children, with a parent with deafblindness, it might be necessary to offer education in sign language for them to be able to better communicate with their parents, as the findings identified that this could be a problem at times. This might be especially important for children of parents whose hearing and vision get worse and who change communication modality from verbal language to sign language, or from visual sign language to tactile sign language.

The first step in support is to identify children whose parent has an illness or disability (Gullbrå et al., 2014). According to the Swedish Health Care Act (SFS, 2017:30) children have the right to information and support when their parent is suffering from mental illness, has a serious physical condition or injury, or suffers from substance abuse. In such cases, the health care professionals should ask patients if they have children who may be in need of information or support. However, this does not include children whose parent has deafblindness. Thomas et al. (2003) highlighted how important it is for children as relatives to get support from different professional caregivers such as the health care and social services. However, for children of parents with deafblindness it could be that they might also be in need of educational support to do some of their homework, or that some children might need emotional support. Perhaps the teacher or school health nurse could determine if the school-aged children of parents having deafblindness are in need of any support, as they are part of the child’s everyday life. It has been found that children value a health dialogue with the school health nurse, as it give them an opportunity to discuss their own health and situation (Granrud et al., 2020; Rising Holmström & Boström, 2021). However, collaboration with other professionals in school are important for the school health nurses to identify and support children having problems (Granrud et al., 2019).

To summarize, we use the terms of Joseph et al. (2020) who proposed that caring could be conceptualized as three concentric circles. The largest is young people who “care about”, those who are helping in at least some way with household chores, but not to a greater extent than their friends. That is how the majority of the children who participated in this study viewed their life; they lived a family life like everyone else. The next circle, according to Joseph et al. (2020) is the young people who care for someone, who have taken on a level of responsibility that involves household chores, but also more specialist roles, but not to such an extent that it interferes excessively with their social and educational activity. The narratives about adjusting to parental needs, being there for their parent, helping and protecting them from harm identified in the present study could be interpreted as belonging to that circle. Finally, Joseph et al. (2020) describe the third circle, where they place the young people who themselves need care, i.e., those who have taken on caring activities to a much higher degree than their friends. These activities and emotional work prevents the young person from engaging in social and educational activities. In the present study there were only a few accounts which could tentatively belong to this last circle. According to Joseph et al. (2020), each group has its own distinctive needs. Recognizing this, support could be more tailored.

### Strengths and limitations

There are both strengths and limitations of our study that should be highlighted. Interviewing both boys and girls from different ages contributed to rich descriptions, enhancing the credibility of the results (Patton, 2015). Another aspect of credibility was the interactive process between all authors involved discussing the emergent findings from analysis until consensus was reached (Patton, 2015). The credibility of the results was further enhanced by citing examples of how the analysis developed from the exact words from the interviews which were merged into codes, subcategories, categories and an overarching theme, and by citing representative quotations to allow the reader to judge whether the analysis was reasonable in relation to the raw data (Shenton, 2004). In the research group there were mixed pre-understandings: three of the authors (AAC, KH, MB) were paediatric nurses and researchers, experienced in qualitative methods, but at the time of the interviews had limited experience of deafblindness. Another researcher (MW) had a background as a social worker and extensive experience in the deafblind field, and one (ASS) had experience of conducting qualitative analysis but limited experience in the deafblind area. Three of the authors were senior researchers which, together with the multidisciplinary roles of the researchers in our research group, may be a strength of the study, increasing the credibility of the results (Shenton, 2004).

To be consistent when collecting data and thereby increasing the dependability of the results, an interview guide was used, and in the method section the data collection and analysis process was described in detail. It is important that the results reflect the informants’ experiences and not the researchers’ perceptions, and to this end, the use of recordings increased the confirmability of the results (Shenton, 2004); however, determining the transferability of the results rests with the reader. The patterns identified from the interviews depend on the context, which in turn influences the transferability of the results (Lincoln & Guba, 1985). Demographic data were therefore provided so that the reader can draw conclusions on similarities between the study context and that to which the results are to be transferred. However, the details of the demographic data are somewhat limited in order to ensure the confidentiality of the participants, as they belong to a small, and thus easily recognizable population.

## Conclusion and clinical implications

This study is unique as these children, as relatives of parents with deafblindness, have not been given a voice before. The children experienced that they lived an ordinary life like their friends, but it was a different ordinary life. With regard to the family as a system it is important that professionals take the child into account when support is given to the parent with deafblindness and the whole family. This could help the child to feel seen and heard and may promote health.
